# The OlympiAD trial: who won the gold?

**DOI:** 10.3332/ecancer.2017.ed75

**Published:** 2017-12-06

**Authors:** Bishal Gyawali

**Affiliations:** Institute of Cancer Policy, King’s College London, Guy’s Hospital Campus, Great Maze Pond Road, London SE1 9RT, UK.

**Keywords:** PARP, olaparib, BRCA, breast cancer, surrogate

## Abstract

OlympiAD was a phase 3 randomized controlled trial of a PARP inhibitor olaparib for metastatic HER2 negative breast cancer patients harboring a BRCA mutation. Although the OlympiAD trial met its primary endpoint, there are concerns regarding whether olaparib truly improves meaningful outcomes for these patients. In this editorial, I examine these issues in detail. An exploration of these issues will provide important educational insights for oncologists and cancer policy makers. I conclude that although olaparib seems to have won the Gold with OlympiAD, the patients probably have not. We need to stop celebrating a gold-plated bronze as a true gold so that one day our patients can finally get the gold they deserve.

Patients with a germline mutation in the tumour suppressor BRCA gene are at increased risk of developing breast cancer that is HER2 negative. These patients also tend to develop breast cancer at a relatively younger age. The mutation in BRCA also makes these patients susceptible to other cancers such as ovarian cancer. Better therapies for this subgroup of patients are urgently needed. One such therapy showing promise is the use of a PARP inhibitor, which is a targeted therapy aiming at inhibiting the repair of DNA breaks which ultimately leads to death of cancer cells. Because BRCA mutation renders the PARP family of enzymes active leading to repair of DNA breaks and preventing death of cancer cells, PARP inhibitors seem to be an ideal targeted approach for breast cancer patients with BRCA mutation. Indeed, PARP inhibitors such as olaparib and niraparib have been already approved for BRCA mutation positive ovarian cancer. In breast cancer, however, no success story for the PARP inhibitors had been achieved yet.

OlympiAD was a phase 3 randomized controlled trial (RCT) of a PARP inhibitor olaparib for metastatic HER2 negative breast cancer patients harboring a BRCA mutation [1]. Patients who had taken up to 2 previous lines of therapy were randomized 2:1 to olaparib or a drug of the physician’s choice. The primary endpoint was progression-free survival (PFS). The trial met its primary endpoint and improved median PFS by 2.8 months (7.0 v 4.2 months) with a hazard ratio of 0.58 (95% CI 0.43 to 0.80). This trial was presented at the 2017 ASCO plenary and simultaneously published in NEJM [1]. Surely, olaparib seems to have won the gold with the OlympiAD; but will patients actually benefit substantially from this drug? In this editorial, I try to answer this question because in cancer care, it’s always the patients’ win that matters the most.

Although the OlympiAD trial met its primary endpoint, there are concerns regarding whether olaparib truly improves meaningful outcomes for these patients. The biggest issue is with the trial’s primary endpoint: PFS. PFS is sometimes acceptable as a surrogate endpoint when a) the survival is good and therefore, it takes a very long time to get the overall survival (OS) results and b) it correlates well with OS. For patients with metastatic breast cancer that are HER2 negative who have already progressed on up to 2 previous lines of therapy, the OS is short. Thus, it is more appropriate to wait for OS results that are more reliable and free of bias. In addition, OlympiAD was an open-label trial without a placebo control. For a trial that is not placebo-controlled, PFS can provide misleading results and OS should be the primary endpoint; especially when measuring OS wouldn’t take as long time as for example in first-line hormone positive breast cancer. It is also interesting to note that the correlation of PFS with OS was low for breast cancer in most meta-analyses [2]. However, whether the PFS benefit correlates with OS is a moot point here because the OS results (not surprisingly) are already available. The OS was similar between the groups: a median of 19.3 versus 19.6 months [1]. The 95% confidence interval for the hazard level of the OS spans from 0.63 to 1.29, meaning by the data are compatible with not only a 37% reduction in mortality but also a 29% increase in mortality. Additionally, because the OS is short, prolonging PFS alone without impacting OS has lesser significance to the patients. Also, precisely because the OS is short, there is lesser chance of confounding by dramatic efficacy of post-progression therapies to negate the benefit obtained in PFS. In OlympiAD, crossover upon progression was not allowed so crossover cannot explain the lack of benefit in survival either. In addition, the absolute benefit observed in median PFS is not dramatic but a mere 2.8 months. Although this small benefit in PFS might look encouraging considering the fact that a previous PARP inhibitor iniparib had failed in a similar setting in a phase 3 RCT, iniparib was not a real inhibitor of PARP [3]. Taken together, olaparib in the OlympiAD trial doesn’t seem to improve the outcomes of real meaning to the patients.

In OlympiAD, concerns also exist with the control arm employed. Carboplatin has emerged as one of the effective therapies for BRCA mutation positive breast cancers based on RCTs showing improved pCR in neoadjuvant setting with the addition of carboplatin [4]. Because PARP inhibitors induce synthetic lethality in tumours with BRCA mutations not only by interfering with DNA repair but also by causing direct toxicity to DNA through PARP trapping [5], it is unsurprising that carboplatin, also a DNA damaging agent, has shown efficacy results in a phase 3 RCT in first-line setting similar to that observed with olaparib in OlympiAD in BRCA mutation positive breast cancer: a response rate of 68% (versus 59.9% in OlympiAD) and PFS of 6.8 months (versus 7 months in this trial) [6]. Also, another PARP inhibitor veliparib failed to improve PFS in a phase 2 RCT in combination with carboplatin and paclitaxel further questioning the efficacy of PARP inhibitors when carboplatin is given [7]. Thus, carboplatin as a control arm would have been the ideal control to test the efficacy of olaparib. However, this trial used a control of physician’s choice which was not a true choice because the use of carboplatin was specifically disallowed. The choice available was capecitabine, eribulin or vinorelbine, but not carboplatin. Although previous treatment with carboplatin was allowed, only 14% of patients had received carboplatin previously for metastatic breast cancer. In fact, subgroup analysis shows that the benefit in PFS was not as substantial for the patients who had previously taken platinum. However, being a subgroup analysis, this can only be considered a hypothesis.

And yet, despite all these caveats, olaparib failed to improve survival.

It is noteworthy that olaparib did have lower toxicities and improved quality of life compared to control. However, when the comparator is 3 different drugs, drawing conclusions is difficult because these 3 drugs differ widely in their toxicities and the number of patients is small in each drug cohort. Considering the toxicities of eribulin, vinorelbine and capecitabine together for overall toxicity or quality of life data is a good real-world example of the so-called “apples and oranges”. Moreover, as argued earlier, the ideal real world comparator would have been carboplatin and whether the toxicities and quality of life parameters would differ compared to carboplatin remains unknown.

In addition, two socio-economic and ethical issues beg discussion with regards to olaparib and the OlympiAD trial. Olaparib is an expensive drug with a hefty monthly price tag of $12,155 (https://www.mskcc.org/sites/default/files/node/25097/documents/111516-drug-costs-table.pdf). In this trial, the median total treatment duration with olaparib was 8.2 months; which equates to a treatment cost of nearly $100,000 for drug alone. For a 2.8 months benefit in PFS without benefit in OS observed in an unblind trial against a weak comparator, are we ready as a society to pay $100,000 per patient? Indeed, it is difficult to define value for cancer drugs; [8] but if $100,000 can be charged without improving survival, would there be any incentives to work harder to improve real significant outcomes for our patients?

Finally, the Trial Oversight section of the article reads “Astrazeneca was responsible for overseeing the collection, analysis, and interpretation of the data….The manuscript was written with medical-writing support, which was funded by Astrazeneca, with critical review and input from authors” [1]. There is no debate as to the importance of collaboration between the industry and clinicians to conduct trials but shouldn’t we draw a line somewhere in this relationship so as to allow the public to trust the data presented? In my opinion, at the very least, the “interpretation” and “writing of manuscript” part of any trial should be exclusively the responsibility of clinicians involved in the trials, and not the sponsor.

## Conclusion

In conclusion, although olaparib seems to have won the Gold with OlympiAD, patients probably have not. Patients deserve a real gold. We need to stop celebrating a gold-plated bronze as a true gold so that one day our patients can finally get the gold they deserve.
